# Mead acid inhibits retinol-induced irritant contact dermatitis via peroxisome proliferator-activated receptor alpha

**DOI:** 10.3389/fmolb.2023.1097955

**Published:** 2023-02-07

**Authors:** Azusa Saika, Prabha Tiwari, Takahiro Nagatake, Eri Node, Koji Hosomi, Tetsuya Honda, Kenji Kabashima, Jun Kunisawa

**Affiliations:** ^1^ Laboratory of Vaccine Materials, Center for Vaccine and Adjuvant Research and Laboratory of Gut Environmental System, Collaborative Research Center for Health and Medicine, National Institutes of Biomedical Innovation, Health and Nutrition (NIBIOHN), Ibaraki, Osaka, Japan; ^2^ Laboratory for Transcriptome Technology, RIKEN Center for Integrative Medical Sciences, Yokohama, Kanagawa, Japan; ^3^ Laboratory of Functional Anatomy, Department of Life Sciences, School of Agriculture, Meiji University, Kawasaki, Kanagawa, Japan; ^4^ Department of Dermatology, Hamamatsu University School of Medicine, Hamamatsu, Shizuoka, Japan; ^5^ Department of Dermatology, Graduate School of Medicine, Kyoto University, Kyoto, Kyoto, Japan; ^6^ International Vaccine Design Center, The Institute of Medical Science, The University of Tokyo, Minato, Tokyo, Japan; ^7^ Graduate School of Medicine, Graduate School of Dentistry, Graduate School of Pharmaceutical Sciences, Graduate School of Science, Osaka University, Suita, Osaka, Japan; ^8^ Department of Microbiology and Immunology, Graduate School of Medicine, Kobe University, Kobe, Hyogo, Japan; ^9^ Research Organization for Nano and Life Innovation, Waseda University, Shinjuku, Tokyo, Japan; ^10^ Graduate School of Biomedical and Health Sciences, Hiroshima University, Higashi-Hiroshima, Hiroshima, Japan

**Keywords:** mead acid, retinol, irritant contact dermatitis (ICD), lipid metabolite, keratinocyte, hyperproliferation, inflammation, oleic acid

## Abstract

Retinol is widely used in topical skincare products to ameliorate skin aging and treat acne and wrinkles; however, retinol and its derivatives occasionally have adverse side effects, including the induction of irritant contact dermatitis. Previously, we reported that mead acid (5,8,11-eicosatrienoic acid), an oleic acid metabolite, ameliorated skin inflammation in dinitrofluorobenzene-induced allergic contact hypersensitivity by inhibiting neutrophil infiltration and leukotriene B_4_ production by neutrophils. Here, we showed that mead acid also suppresses retinol-induced irritant contact dermatitis. In a murine model, we revealed that mead acid inhibited keratinocyte abnormalities such as keratinocyte hyperproliferation. Consistently, mead acid inhibited p38 MAPK (mitogen-activated protein kinase) phosphorylation, which is an essential signaling pathway in the keratinocyte hyperplasia induced by retinol. These inhibitory effects of mead acid were associated with the prevention of both keratinocyte hyperproliferation and the gene expression of neutrophil chemoattractants, including Cxcl1 and Cxcl2, and they were mediated by a PPAR (peroxisome proliferator-activated receptor)-α pathway. Our findings identified the anti-inflammatory effects of mead acid, the use of which can be expected to minimize the risk of adverse side effects associated with topical retinoid application.

## Introduction

Retinol is commonly applied topically in various skin disorders such as psoriasis and ichthyosis and is widely used in skincare products to ameliorate skin aging and treat acne, wrinkles, and warts (Kafi et al., 2007; Lee et al., 2009; Al Tanoury et al., 2013; Sadick et al., 2019; Szymanski et al., 2020). However, many people receiving topical retinoids such as retinol, retinoic acid, and their derivatives develop an irritant contact dermatitis (ICD), known as retinoid-induced dermatitis, which is characterized by erythema, scaling, dryness, burning, and pruritus. These undesirable side effects cause some people to discontinue treatment (David et al., 1988; Kim et al., 2003; Buchanan and Gilman, 2016). ICD is generally described as a multifactorial disorder that occurs as a direct response to chemical properties such as alkalinity or acidity. However, the mechanisms behind retinoid-induced ICD are considered to be complex and to differ from those of other types of irritation: The irritation induced by retinoids is mediated by receptors, including retinoic acid receptors (RARs) and retinoid X receptors (RXRs) (Matsunaga et al., 2012; Szymanski et al., 2020).

In retinoid-induced dermatitis, heparin-binding epidermal growth factor-like growth factor (HB-EGF) is a major paracrine factor synthesized in keratinocytes through RAR and RXR activation. It mediates epidermal hyperplasia, which is a characteristic feature of retinoid dermatitis (Stoll and Elder, 1998). Marked HB-EGF gene expression is induced by retinoic acid treatment in human and murine skin, and there is a positive correlation between this expression and hyperproliferation of keratinocytes (Xiao et al., 1999; Yoshimura et al., 2003). In addition, a study in hairless mice has shown that increased levels of HB-EGF and epidermal hyperplasia are more prominent features in retinol-induced ICD than in non-receptor-mediated ICD induced by benzalkonium chloride (Lee et al., 2010). Therefore, epidermal hyperplasia and elevated HB-EGF are recognized as major features of retinoid-induced dermatitis.

Dietary fatty acids affect immunological functions, including inflammatory and allergic responses; it is generally accepted that metabolites derived from omega-3 polyunsaturated fatty acids, which have a double bond in the third position from the methyl end, work as functional anti-inflammatory mediators to resolve inflammatory reactions (Cucchi et al., 2019). We previously reported that metabolites derived from the omega-3 fatty acid eicosapentaenoic acid, such as 12-hydroxyeicosapentaenoic acid, 15-hydroxyeicosapentaenoic acid, 17,18-epoxyeicosatetraenoic acid, and 14-hydroxyeicosapentaenoic acid, showed anti-inflammatory or anti-allergic activity, or both, *via* a variety of mechanisms (Kunisawa et al., 2015; Nagatake et al., 2018; Sawane et al., 2019; Saika et al., 2021). Several fatty acid metabolites derived from omega-3 fatty acids have emerged as promising candidates for treating various inflammatory diseases (Herrera Vielma et al., 2021). In addition, the biological functions of omega-9 mono- and polyunsaturated fatty acids, such as oleic acid, mead acid, and elaidic acid, have recently received wide attention in response to emerging studies and discoveries revealing their biological benefits (Farag and Gad, 2022). Mead acid (5,8,11-eicosatrienoic acid) is a functional polyunsaturated omega-9 fatty acid derived from oleic acid (Gramlich et al., 2019). Mead acid inhibits the development and progression of mammary carcinogenesis by suppressing the proliferation of cancer cells, including the human breast cancer cell lines MCF-7 and KPL-1 (Kinoshita et al., 2014). In inflammatory diseases, supplementation with mead acid has therapeutic effects on indomethacin-induced bowel lesions by suppressing leukotriene B_4_ (LTB_4_) synthesis (Yoshida et al., 2003). We found previously that intraperitoneal injection of mead acid ameliorates skin inflammation in a murine model of dinitrofluorobenzene (DNFB)-induced allergic contact hypersensitivity by inhibiting neutrophil migration into the site of inflammation and suppressing an increase in vascular permeability (Tiwari et al., 2019). Furthermore, we showed that mead acid inhibits the secondary influx of neutrophils by suppressing their LTB_4_ production (Tiwari et al., 2019). However, the detailed molecular mechanisms and the functional receptor responsible for these anti-inflammatory effects are still unknown.

Here, we used a retinol-induced ICD murine model to investigate the potential roles of mead acid in inhibiting the side effects of retinoid therapy, and we confirmed the regulatory activity of mead acid on keratinocytes. We found that mead acid ameliorated skin inflammation in retinol-induced ICD through a peroxisome proliferator-activated receptor (PPAR)-α-mediated pathway by inhibiting keratinocyte abnormalities such as keratinocyte hyperplasia and the gene expression of neutrophil chemoattractants. In addition, we found that mead acid inhibited the phosphorylation of p38 mitogen-activated protein kinase (MAPK), which is an essential signaling pathway that enhances the keratinocyte abnormalities induced by retinol.

## Materials and methods

### Mice

Before the experiments, female BALB/c mice (6-7 weeks old) were purchased from Japan SLC (Hamamatsu, Japan) and maintained in a specific-pathogen-free animal facility at NIBIOHN (National Institutes of Biomedical Innovation, Health and Nutrition, Ibaraki, Osaka, Japan). Mice were euthanized by cervical dislocation under anesthesia with isoflurane (AbbVie, North Chicago, Illinois, United States). All experiments were conducted in accordance with the guidelines of the Animal Care and Use Committee and the Committee on the Ethics of Animal Experiments at NIBIOHN.

### Retinol-induced ICD murine model

Retinol-induced ICD was induced as described previously, with some modifications (Lee et al., 2010; Kim et al., 2012). Briefly, both ears of each mouse were treated topically once daily for 4 consecutive days with 10 µL of 0.05% (w/v) all-trans-retinol (Sigma-Aldrich, St. Louis, Missouri, United States) dissolved in 4:3:3 dimethyl acetamide (Sigma-Aldrich), acetone (Nacalai Tesque, Kyoto, Japan), and ethanol (Nacalai Tesque). Mead acid (10 µg/10 μL, Cayman Chemical, Ann Arbor, Michigan, United States), oleic acid (10 µg/10 μL, Cayman Chemical), or vehicle control 3:1 ethanol in phosphate-buffered saline (PBS) was applied topically to both sides of the ears 45 min after each of the inflammatory insults. Ear thickness was evaluated with a micrometer MDC-25MJ 293–230 (Mitsutoyo, Kawasaki, Japan).

In experiments using receptor antagonists, GSK0660 (100 μg/10 μL, Sigma-Aldrich), GW6471 (100 μg/10 μL, Sigma-Aldrich), or vehicle control 1:1 dimethyl sulfoxide (DMSO, Nacalai Tesque) and 50% (v/v) ethanol in PBS was applied topically to both sides of the ears. Thirty minutes after treatment with the antagonist, mead acid or vehicle control was applied topically, and 45 min later retinol was applied topically at the dose rate described above. These treatment sequences were performed once daily for 4 consecutive days. In experiments using kinase inhibitors, SB202190 (Sigma-Aldrich), or U1026 (Sigma-Aldrich), or SP600125 (Sigma-Aldrich), or vehicle control 1:1 DMSO and 50% (v/v) ethanol in PBS were applied at 20 mM/10 μL topically on both sides of the ears 45 min after topical application of the retinol at the dose rate described above. These treatment sequences were continued once daily for 4 consecutive days. Ear swelling (Δµm) was calculated as (Ear thickness [µm] on the indicated day)—(Ear thickness [µm] before the first retinol application).

### Cell isolation and flow cytometric analysis

Cell isolation and flow cytometry were performed as described previously (Nagatake et al., 2018). In brief, murine ear samples were digested, with stirring, for 60 or 90 min at 37°C with 2 mg/mL collagenase (Wako Pure Chemicals, Osaka, Japan) in RPMI 1640 medium (Sigma-Aldrich) containing 2% (v/v) newborn calf serum (Equitech-Bio, Kerrville, Texas, United States). We treated the samples with collagenase for 90 min to analyze immune cells and for 60 min to sort keratinocytes. Cell suspensions were filtered through a 70-µm cell strainer (BD Biosciences, Franklin Lakes, New Jersey, United States) and stained with FITC (fluorescein isothiocyanate)–anti-mouse-Ly6G antibody (BioLegend, San Diego, California, United States; 1:100), APC (allophycocyanin)–Cy7 anti-CD11b antibody (BioLegend; 1:100), PE (phycoerythrin)–anti-CD31 antibody (BD Biosciences; 1:100), FITC–anti-CD34 antibody (BD Biosciences; 1:100), APC–anti-CD49f antibody (BioLegend; 1:100), PE–anti-Ki-67 antibody (BioLegend; 1:100), or BV (Brilliant Violet)-421–anti-CD45 antibody (BioLegend; 1:100). Murine keratinocytes were isolated as the CD45^–^ CD31^–^ CD34^–^ CD49f^+^ cell fraction, as described previously (Saika et al., 2021). Samples were analyzed by using a MACSQuant (Miltenyi Biotec, Bergisch Gladbach, Germany) or FACSAria (BD Biosciences) cell sorter, and cells were isolated with a FACSAria. Data were analyzed by using Flowjo 9.9 (Tree Star, Ashland, Oregon, United States).

### Histological analysis

Histological analysis was performed as described previously (Nagatake et al., 2018). The mice were sacrificed on day 4 of the experiment, after which ear samples were collected immediately. Frozen tissue sections (6 μm thick) were prepared by cryostat (Leica, Wetzlar, Germany, CM3050S) and used for hematoxylin and eosin staining and immunohistological analysis. The following antibodies were used in the immunohistological analysis: anti-cytokeratin 10 (K10) antibody (Abcam, Cambridge, United Kingdom), FITC–anti-Ly6G antibody, PE–anti-Ki-67 antibody, and APC–anti-CD49f antibody. AF (Alexa-Fluor)-488–anti-rabbit IgG (Thermo Fisher Scientific, Waltham, Massachusetts, United States) was used as a secondary antibody to detect K10. Cell nuclei were stained with 4′,6-diamidino-2-phenylindole (DAPI, AAT Bioquest, Sunnyvale, California, United States). Tissue sections were examined under a fluorescence microscope (model BZ-9000, Keyence, Osaka, Japan). The staining area of images was analyzed by using the Analyzer software for the BZ-X700 (Keyence).

### Reporter assays

The Indigo Biosciences (State College, Pennsylvania, United States) Reporter Assay System was used for the luciferase reporter assay. Fatty acids were tested for their ability to activate nuclear receptors by using human PPARα and PPARβ reporter assay systems (Indigo Biosciences) followed by the procedure described in the kits. In brief, reporter cells expressing a hybrid receptor composed of the Gal4 DNA-binding domain fused to the ligand-binding domain of the specific nuclear receptor, together with the firefly luciferase reporter gene, were provided with the reporter assay systems. Several concentrations (0.3, 3, and 30 µM) of mead acid and oleic acid were used for the assay. Oleic acid was used as a control for mead acid. After 24 h of incubation (37°C in 5% CO_2_) of these mixtures with the tested compounds, the activities of the nuclear receptors were quantified as relative light units by measuring the emission of light with a microplate luminometer (Arvo X2, Perkin Elmer, Waltham, Massachusetts, United States).

### Cell culture

HaCaT cells (immortalized human keratinocytes) were obtained from CLS Cell Lines Service (Eppelheim, Germany) (Boukamp et al., 1988) and cultured as described previously (Saika et al., 2021). Cultured HaCaT cells were seeded at a concentration of 5 × 10^5^ cells/10 mL in Dulbecco’s modified Eagle’s medium high glucose (Sigma-Aldrich) supplemented with 10% fetal bovine serum (FBS, Gibco) and 100 U/mL penicillin with 100 μg/mL streptomycin (Nacalai Tesque) and incubated for 24 h at 37°C in 5% CO_2_. The medium was then replaced with Dulbecco’s modified Eagle’s medium without FBS, and the cells were treated with 3 µM mead acid or vehicle (ethanol) for 30 min before stimulation with HB-EGF (1 ng/mL, PeproTech, Cranbury, New Jersey, United States) for 1 h at 37°C in 5% CO_2_.

### Western blot analysis

HaCaT cells were harvested by being scraped in 300 µL of RIPA (Radioimmunoprecipitation Assay) lysis buffer (EMD Millipore Corporation, Burlington, Massachusetts, United States) containing protease cocktail inhibitor (Sigma-Aldrich) and PhosSTOP phosphatase inhibitor (Roche, Basel, Switzerland). The protein in the samples was quantified by using a BCA (bicinchoninic acid) protein assay kit (Thermo Fisher Scientific) in accordance with the instructions in the kit. An equal amount of protein was separated on NuPAGE 4%–12% Bis-Tris gels (Thermo Fisher Scientific), and transferred to polyvinylidene difluoride membranes (EMD Millipore Corporation). The antibodies used were anti-phospho-p38 MAPK and anti-p38 MAPK polyclonal antibodies (Cell Signaling, Danvers, Massachusetts, United States) and horseradish-peroxidase-conjugated donkey anti-rabbit IgG (BioLegend). Western blot signals were analyzed by using an image analyzer (LAS-4000, ImageQuant, GE Healthcare, Tokyo, Japan).

### Gene expression analysis

After daily application of retinol or vehicle for 4 days, ear samples were harvested in XXTuff microvials (BioSpec Products, Bartlesville, Oklahoma, United States), disrupted by using stainless-steel beads (φ 48 × 1, φ 32 × 4, TOMY, Tokyo, Japan) at 4,800 rpm for 10 s, and then pulsed five times by using a tissue homogenizer (Precellys 24, Bertin Instruments, Montigny-le-Bretonneux, France). Total RNA was isolated by using the ReliaPrep RNA Tissue Miniprep System (Promega, Madison, Wisconsin, United States). In some experiments, keratinocytes were isolated from ear samples; total RNA was prepared by using Sepazol reagent (Nacalai Tesque) and chloroform (Nacalai Tesque), precipitated with 2-propanol (Nacalai Tesque), washed with 75% (v/v) ethanol (Nacalai Tesque), and treated with DNase Ι (Promega).

cDNA was prepared from samples by using a cDNA Synthesis Kit (Invitrogen, Carlsbad, California, United States). Quantitative RT-PCR analysis was performed with a LightCycler 480 II PCR platform (Roche) with FastStart Essential DNA Probes Master reaction mix for PCR (Roche) or SYBR Green I Master reagents (Roche).

Gene expression levels were normalized to that of the β-actin gene *Actb*. The following primer sequences were used for PCR with FastStart Essential DNA Probes Master for mouse: *Cxcl1* forward, 5′-gac​tcc​agc​cac​act​cca​ac-3´; *Cxcl1* reverse, 5′-tga​cag​cgc​agc​tca​ttg-3′; *Cxcl2* forward, 5′-aaa​atc​atc​caa​aag​ata​ctg​aac​aa-3′; *Cxcl2* reverse, 5′-ctt​tgg​ttc​ttc​cgt​tga​gg-3′; *Hbegf* forward, 5′-tct​tct​tgt​cat​cgt​ggg​act-3′; *Hbegf* reverse, cac​gcc​caa​ctt​cac​ttt​ct-3′; *Actb* forward, 5′-aag​gcc​aac​cgt​gaa​aag​at-3′; and *Actb* reverse, 5′-gtg​gta​cga​cca​gag​gca​tac-3′. The following primer sequences were used for PCR with SYBR Green I Master reagents for human: *DUSP1* forward, 5′-agt​acc​cca​ctc​tac​gat​cag​g-3′; *DUSP1* reverse, 5′-gaa​gcg​tga​tac​gca​ctg​c-3′; *DUSP6* forward, 5′-gaa​atg​gcg​atc​agc​aag​acg-3′; *DUSP6* reverse, 5′-cga​cga​ctc​gta​tag​ctc​ctg-3′; *MKK3* forward, 5′-gac​tcc​cgg​acc​ttc​atc​ac-3′; *MKK3* reverse, 5′-ggc​cca​gtt​ctg​aga​tgg​t-3′; *MKK4* forward, 5′-tgc​agg​gta​aac​gca​aag​ca-3′; *MKK4* reverse, 5′-ctc​ctg​tag​gat​tgg​gat​tca​ga-3′; *ACTB* forward, 5′- cat​gta​cgt​tgc​tat​cca​ggc-3′; and *ACTB* reverse, 5′-ctc​ctt​aat​gtc​acg​cac​gat -3´.

### Analysis of cell viability

Cell viability was calculated by using a CellTiter 96 Non-Radioactive Cell Proliferation Assay kit (Promega) in accordance with the manufacturer’s instructions. Briefly, HaCaT cells were seeded at 1 × 10^4^ cells/well on 96-well plates and then incubated for 24 h at 37°C in 5% CO_2_. Mead acid or oleic acid was added, and the plates containing fatty acids in each concentration of 300 pM to 300 μM were incubated for 1 h or 24 h. After each exposure time, the culture medium was removed, and 3-(4,5-dimethyl-2-thiazolyl)-2,5-diphenyltetrazolium bromide dye solution was added to each well. The plates were then incubated for 4 h at 37°C in 5% CO_2_, and solubilization/stop solution was added. After an overnight incubation, the absorbance at OD_570_ was measured by using an iMark microplate reader (Bio-Rad, Hercules, California, United States). Ethanol at 0.2% (v/v) in the culture medium was used as a vehicle control. The percentage of viable cells was calculated by using the following formula: (%) = [100% × (sample OD_570_)/(vehicle control OD_570_)].

### Statistical analysis

Data were analyzed by using the non-parametric Kruskal–Wallis test followed by Dunn’s multiple comparison test, the Mann-Whitney *U*-test, or Welch’s *t*-test (Prism 6, GraphPad Software, San Diego, California, United States). A *p*-value of less than 0.05 was considered significant.

## Results

### Mead acid ameliorates inflammatory signs in a retinol-induced ICD murine model

**FIGURE 1:**
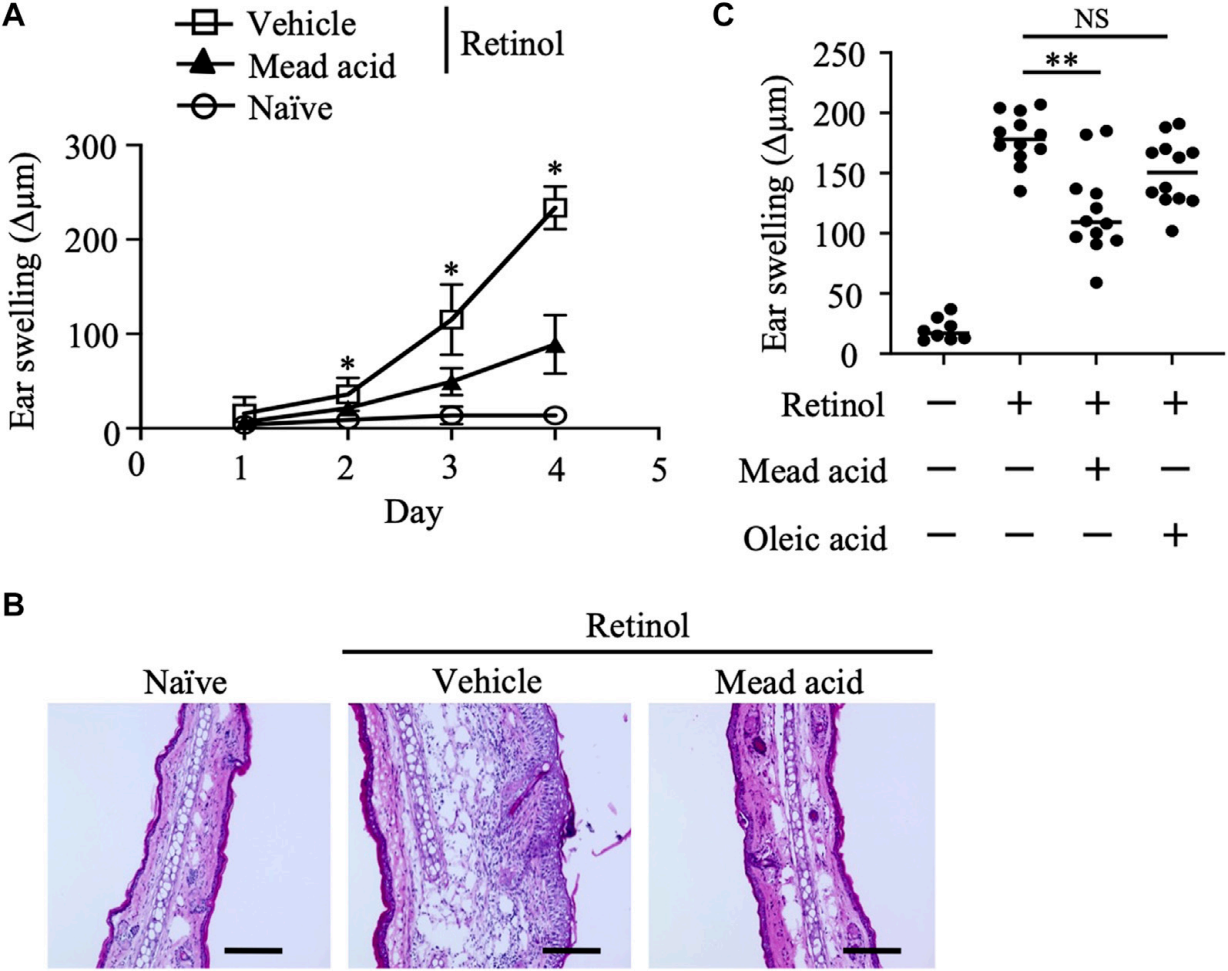
Retinol-induced irritant contact dermatitis (ICD) is inhibited by mead acid treatment. Retinol-induced ICD was induced by topical application of retinol for 4 consecutive days. Mice were treated topically with vehicle or mead acid 45 min after each retinol application. **(A)** Ear thickness was measured daily. Ear swelling (Δµm) was calculated as (Ear thickness [µm] on the indicated day)—(Ear thickness [µm] before the first retinol application). *n* = 8/group. Data were combined from two independent experiments (mean ± SEM values). Significant differences (*p* < 0.05) shown are between the vehicle and mead-acid-treatment groups. **(B)** For histological analysis, frozen ear sections were stained on day 5 with hematoxylin and eosin. Representative images from three independent experiments are shown. Scale bars, 100 μm. **(C)** Ear thickness was measured on day 4. Ear swelling (Δµm) was calculated as (Ear thickness [µm] on day 4)—(Ear thickness [µm] before the first retinol application). Horizontal bars indicate median values. Data are representative of three independent experiments. NS, not significant; **p < 0.01.

Given that topical retinol treatment causes epidermal thickening (Lee et al., 2010; Kong et al., 2016), we first evaluated the inflammatory signs in a murine model of retinol-induced ICD. In this model, retinol was applied topically to both sides of the ear for 4 consecutive days and ear thickness (and hence swelling) was measured on days 1, 2, 3, and 4. Ear swelling increased in a time-dependent manner in the retinol-treated mice but not in the untreated naïve mice (Figure 1A). In addition, on days 2–4, mead acid treatment reduced the ear swelling induced by retinol treatment compared with vehicle treatment (Figure 1A). Histological analysis of ear sections of vehicle-treated mice showed increases in ear thickness, the thickness of the epidermal layer, and cell infiltration into the dermis compared with those in naive mice (Figure 1B). However, these inflammatory signs were ameliorated in ears cotreated with mead acid (Figure 1B). Because mead acid is a metabolite derived from oleic acid, we also examined whether oleic acid also had anti-inflammatory activity, but no anti-inflammatory activity was detected (Figure 1C), indicating that the suppressive effect in retinol-induced ICD was a particular characteristic of the mead acid treatment.

### Mead acid inhibits neutrophil recruitment at inflammation site

**FIGURE 2:**
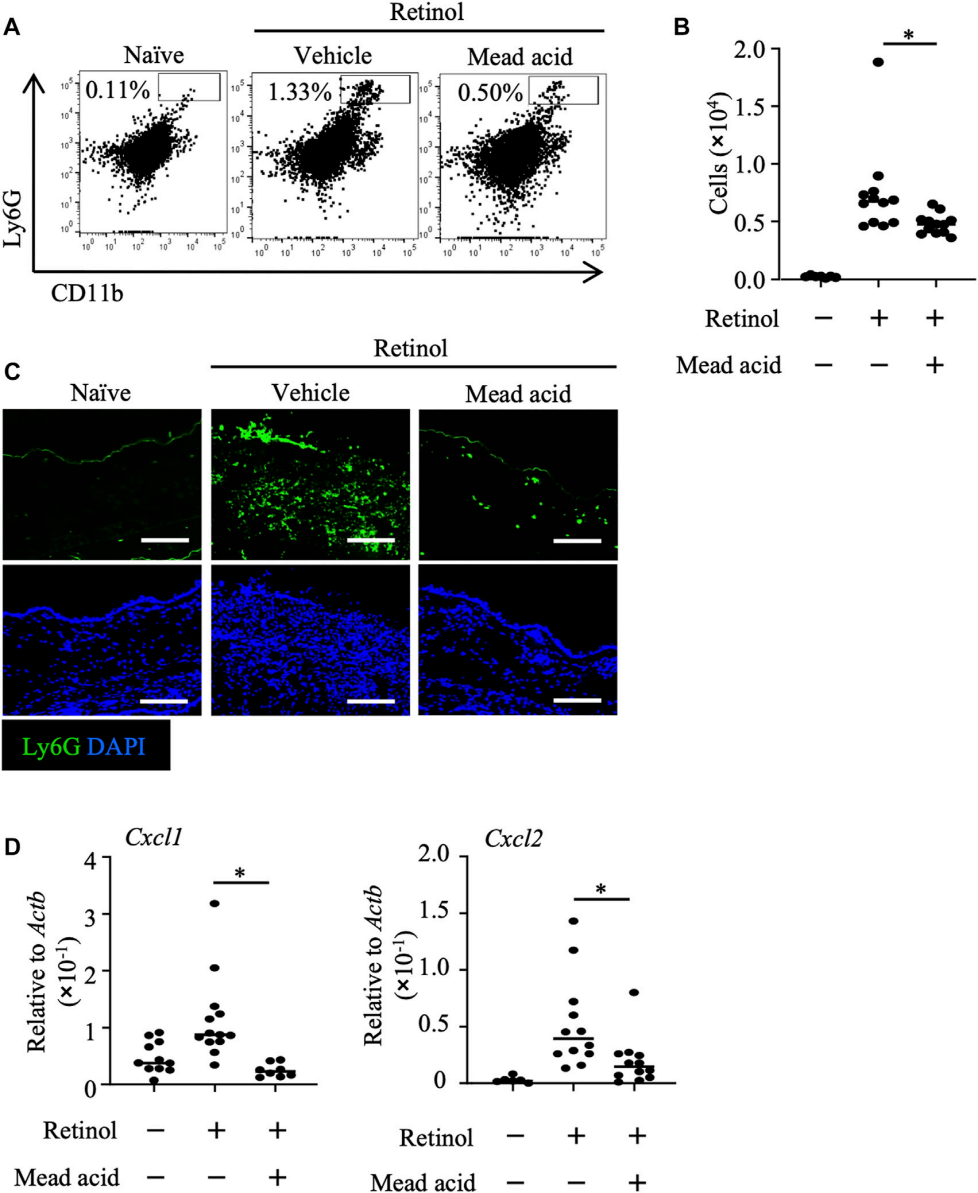
Mead acid inhibits neutrophil infiltration in retinol-induced irritant contact dermatitis (ICD). ICD was induced by topical application of retinol for 4 consecutive days. Mice were treated topically with vehicle or mead acid after each retinol treatment. **(A)** Cells were isolated from murine ears and used for flow cytometric analysis. Numbers indicate the percentages of Ly6G^+^ CD11b^+^ neutrophils. Representative images are shown. **(B)** The number of Ly6G^+^ CD11b^+^ neutrophils was determined on the basis of total cell numbers and flow cytometric data. **(C)** Frozen ear sections obtained on day 4 were stained with the indicated antibodies for immunohistologic analysis. Representative images from two independent experiments are shown. Bars, 100 μm. **(D)** Ear tissues were homogenized to isolate mRNA, and *Cxcl1* and *Cxcl2* expression was measured by quantitative RT-PCR analysis and normalized to that of *Actb*. Data are combined from four independent experiments. Horizontal bars indicate median values. *p < 0.05.

We previously reported that intraperitoneal injection of mead acid inhibited neutrophil chemotaxis into the site of inflammation in a DNFB-induced allergic contact hypersensitivity murine model (Tiwari et al., 2019). In addition, we showed that mead acid acted directly on neutrophils and inhibited neutrophil migration by suppressing their pseudopod formation and LTB_4_ production. Neutrophil accumulation in the dermis has been reported in both retinol dermatitis and DNFB-induced allergic contact hypersensitivity (Varani et al., 2008). In light of our previous findings, we examined whether mead acid regulated neutrophils in retinol-induced ICD. As we showed in our previous report (Tiwari et al., 2019), mead acid reduced the increase in neutrophil numbers in inflamed ear skin (Figures 2A, B). Immunohistological analysis revealed that the abundance of Ly6G^+^ neutrophils in the dermis was lower in the ear sections of mice that had received mead acid co-treatment (Figure 2C). These results indicated that mead acid inhibited neutrophil infiltration into the inflamed skin in retinol-induced ICD.

Neutrophil recruitment is promoted by the indirect mechanisms of neutrophil chemoattractants such as Cxcl1 and Cxcl2 (Saika et al., 2021). The major source of neutrophil-attracting chemokines is keratinocytes (Lee et al., 2013), and neutrophil numbers after induction of inflammation are reduced by inhibition of the expression of neutrophil-attracting chemokines on keratinocytes (Cattani et al., 2006; Saika et al., 2021). Therefore, we analyzed the expression levels of *Cxcl1* and *Cxcl2* on keratinocytes fractionated from murine ears to reveal the indirect neutrophil-regulating activity of mead acid. We found that the increase in expression of *Cxcl1* and *Cxcl2* on keratinocytes sorted from murine ear skin after retinol stimulation was inhibited by mead acid co-treatment (Figure 2D). Thus mead acid inhibited neutrophil infiltration into the inflamed skin by both direct mechanisms, as shown previously (Tiwari et al., 2019), and indirect mechanisms.

### Mead acid inhibits epidermal hyperplasia by inhibiting keratinocyte proliferation

**FIGURE 3:**
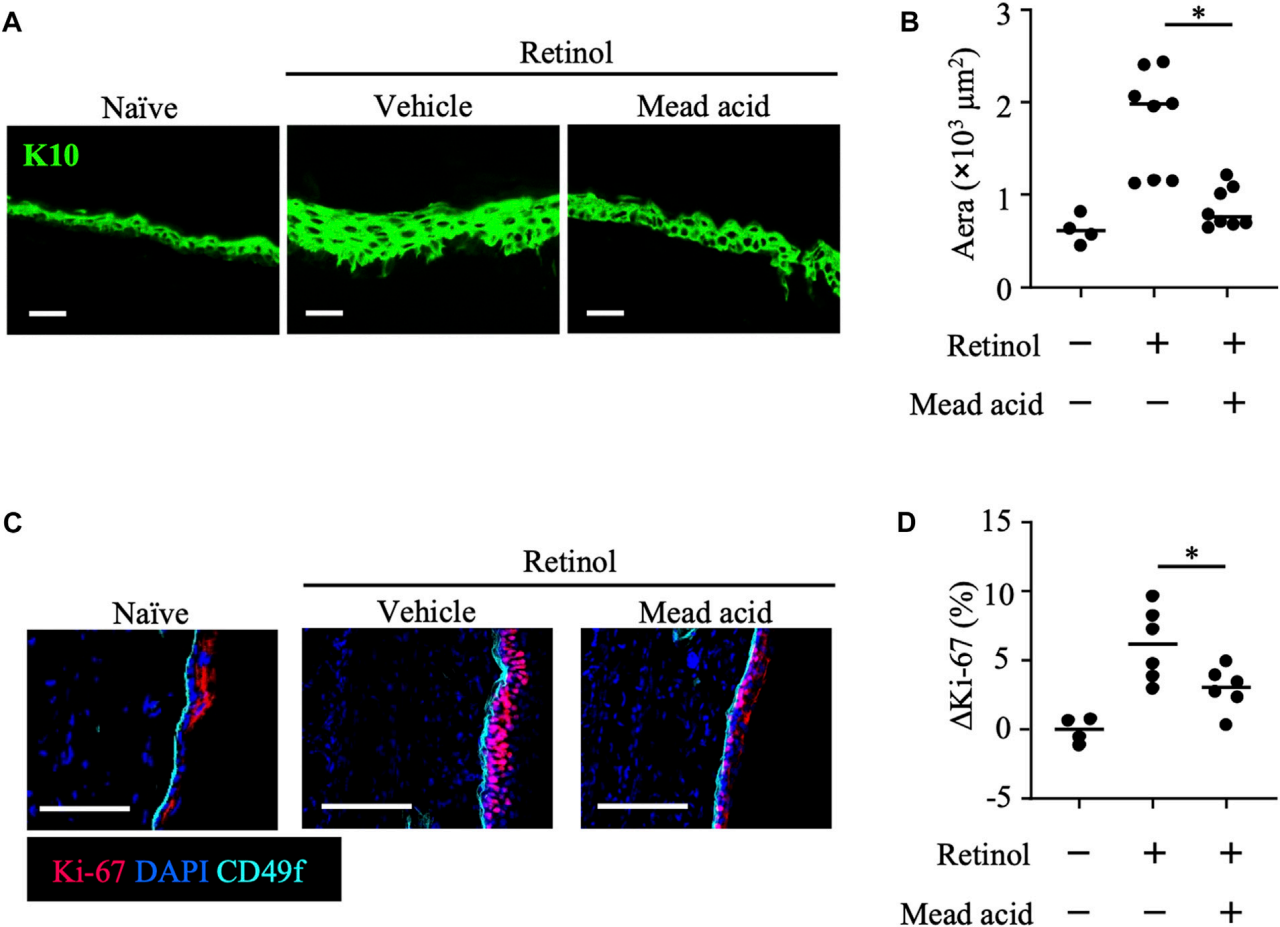
Mead acid inhibits keratinocyte abnormalities induced by retinol. Retinol was applied topically for 4 consecutive days to induce ICD. Mice were treated topically with vehicle or mead acid after retinol treatment. **(A)** Frozen ear sections obtained on day 4 were stained with anti-K10 antibody. Scale bars, 20 μm. Representative images from three independent experiments are shown. **(B)** The area stained for K10 was calculated by using BZ-X700 Analyzer software. **(C)** Frozen ear sections were stained with the indicated antibodies. Representative images from two independent experiments are shown. Bars, 100 μm. **(D)** The percentage of Ki-67-expressing keratinocytes gated as CD45^−^ CD31^−^ CD34^−^ CD49f^+^ was measured by flow cytometry. The rate of increase of Ki-67^+^ cells [ΔKi-67 (%)] was calculated as (Ki-67 [%])—(Ki-67 [%] average in retinol-untreated mice) on day 4. Data are combined from four independent experiments. Horizontal bars indicate median values. *p < 0.05.

In the retinol-induced ICD murine model, epidermal hyperplasia and the consequent increase in local skin thickness are accompanied by the enhancement of keratinocyte proliferation (Chapellier et al., 2002). Because we found that mead acid regulated not only neutrophil function but also keratinocyte function, we used histological analysis to evaluate the degree of epidermal hyperplasia. To this end, we stained ear sections for K10, which is expressed on the stratum spinosum. Whereas retinol treatment increased the thickness of the K10-positive keratinocyte layer, co-treatment with mead acid reduced the thickness of this layer (Figures 3A, B). We next evaluated the expression of Ki-67, a marker of proliferation, in keratinocytes. When we segregated basal keratinocytes by CD49f expression as a marker of epidermal stem cells, Ki-67-expressing cells were predominantly located in the upper part of the basal keratinocyte layer in naïve mice. Retinol treatment increased the numbers of these Ki-67-expressing cells, but mead acid co-treatment decreased the Ki-67-positive cell count (Figure 3C). Flow cytometric analysis confirmed the results by showing that the abundance of Ki-67-positive cells among CD45^–^ CD31^–^ CD34^–^ CD49f^+^ keratinocytes was increased by retinol treatment, whereas the increase was suppressed by mead acid co-treatment (Figure 3D). These results indicated that mead acid inhibited retinol-induced epidermal hyperplasia by inhibiting keratinocyte proliferation.

### Mead acid ameliorates retinol-induced ICD *via* a PPARα-Mediated pathway

**FIGURE 4:**
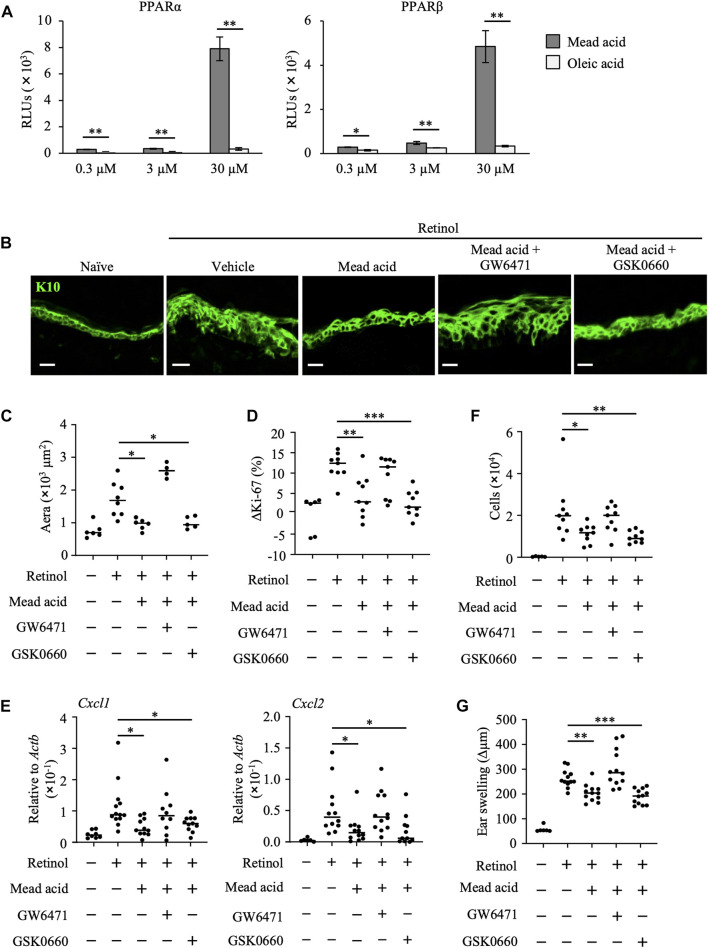
Mead acid reduces retinol-induced irritant contact dermatitis (ICD) *via* a PPARα-mediated pathway. **(A)** Activation levels of PPARα and PPARβ/δ were measured by using a reporter assay system following a 24 h exposure to mead acid or oleic acid (0.3, 3, or 30 µM) or vehicle. Data are combined from two independent experiments (average ±SD values). Statistical significance was determined by using Welch’s *t*-test. **(B–G)** ICD was induced by topical application of retinol. Mice were co-treated with mead acid and a receptor antagonist (GW6471 or GSK0660). **(B)** Frozen ear sections were stained with anti-K10 antibody. Representative images from two independent experiments are shown. Scale bars, 20 μm. **(C)** The area stained for K10 was calculated by using BZ-X700 Analyzer software. **(D)** The percentage of Ki-67-expressing keratinocytes gated as CD45^–^ CD31^–^ CD34^–^ CD49f^+^ was measured by flow cytometry. The rate of increase of Ki-67-positive cells [ΔKi-67 (%)] was calculated as (Ki-67 [%])—(Ki-67 [%] average in retinol-untreated mice) on day 4. Data are combined from four independent experiments. **(E)** Ear tissues were homogenized to isolate mRNA, and *Cxcl1* and *Cxcl2* expression was measured by quantitative RT-PCR analysis and normalized to that of *Actb*. Data are combined from four independent experiments. **(F)** The number of Ly6G^+^ CD11b^+^ neutrophils was determined on the basis of total cell numbers and flow cytometric data. **(G)** Ear thickness was measured on day 4. Ear swelling (Δµm) was calculated as (Ear thickness [µm] on day 4)—(Ear thickness [µm] before the first retinol application). Horizontal bars indicate median values. RLUs, relative light units. *p < 0.05; **p < 0.01; ***p < 0.001.

To identify the functional receptor of mead acid in its anti-inflammatory activities in retinol-induced ICD, we next performed a reporter assay. PPARs are receptors for long-chain free fatty acids and are associated with the reduction of inflammation in conditions such as dermatitis, colitis, inflammatory bowel disease, diabetes, and asthma (Sertznig et al., 2008; Staumont-Sallé et al., 2008; Echeverria et al., 2016). Therefore, we evaluated PPAR activity. We found that, unlike oleic acid, mead acid activated PPARα and PPARβ/δ in a concentration-dependent manner (Figure 4A). We confirmed that mead acid and oleic acid were not toxic to HaCaT cells at any concentration from 300 pM to 300 μM, including the 0.3–30 μM concentrations used in the *in vitro* assay. (Supplementary Figures S1A, B). As mead acid activated PPARγ to almost the same level as those of other free long-chain fatty acids (data not shown), we aimed to evaluate only PPARα and PPARβ/δ to determine whether they acted as functional receptors of mead acid for the amelioration of retinol-induced ICD. To this end, we evaluated the effects of mead acid by topical application of the respective receptor antagonists before mead acid treatment. We found that the inhibitory effect of mead acid on epidermal hyperplasia was cancelled by co-treatment with GW6471 (a PPARα antagonist) but not with GSK0660 (a PPARβ/δ antagonist) (Figures 4B, C). Moreover, the modulating effect of mead acid on the increase in abundance of Ki-67-positive cells among keratinocytes was cancelled by co-treatment with GW6471 but not by treatment with GSK0660 (Figure 4D). In addition, the ability of mead acid to prevent neutrophil infiltration *via* the control of Cxcl1 and Cxcl2 expression on keratinocytes and of neutrophil numbers in inflamed skin was cancelled by co-treatment with GW6471 (Figures 4E, F). Consequently, co-treatment with GW6471 led to a lack of inhibition of ear swelling by mead acid (Figure 4G). These results indicated that mead acid acted as a ligand of both receptors, PPARα and PPARβ/δ, but that PPARα was a functional receptor for its inhibition of skin inflammation in retinol-induced ICD.

### P38 MAPK is a major signaling pathway for induction of keratinocyte abnormalities

Retinol is known to enhance MAPK pathways, which play essential roles in the regulation of cell proliferation and in the production of inflammatory cytokines and chemokines (Yen et al., 1998; Lee et al., 1999; Yu et al., 2003; Lee et al., 2008; Piskunov and Rochette-Egly, 2012). The underlying signaling that accelerates retinoid-induced inflammation is not fully understood. Therefore, by using specific inhibitors, we first evaluated which major signaling pathway, namely p38 MAPK, MAPK/extracellular signal-regulated kinase (ERK), or c-Jun amino-terminal kinase (JNK), was involved in retinol-induced keratinocyte abnormalities.

**FIGURE 5:**
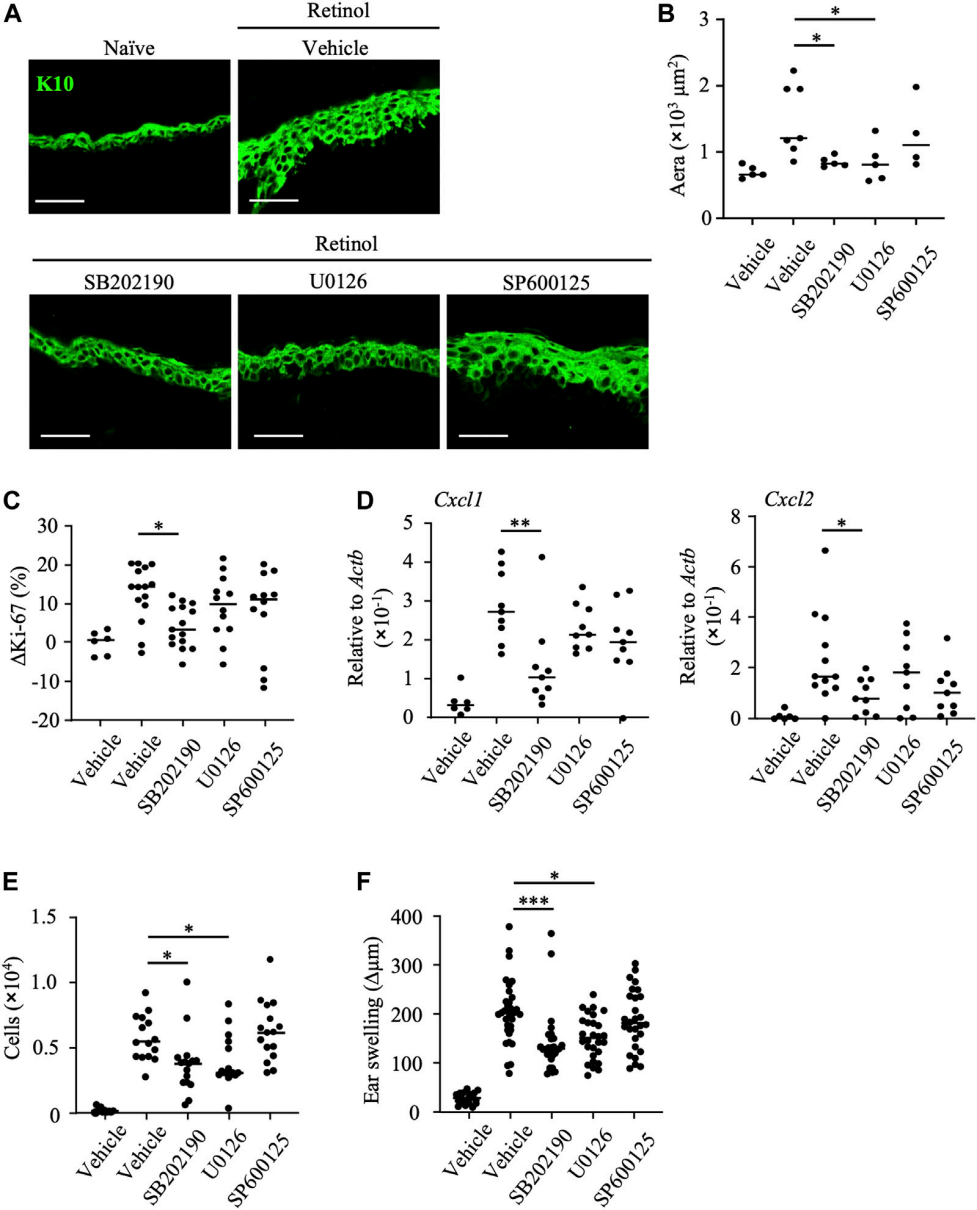
Inhibition of p38 MAPK decreases keratinocyte abnormalities induced by retinol. After topical application of retinol, the p38MAPK inhibitor SB202190, the MAPK/ERK inhibitor U1026, the JNK inhibitor SP600125, or vehicle was applied topically to both sides of mouse ears. **(A)** Frozen ear sections obtained on day 4 were stained with anti-K10 antibody. Representative images from three independent experiments are shown. Scale bars, 20 μm. **(B)** The area stained for K10 was calculated by using BZ-X700 Analyzer software. **(C)** The percentage of Ki-67-expressing keratinocytes gated as CD45^−^ CD31^−^ CD34^−^ CD49f^+^ was measured by flow cytometry. The rate of increase of Ki-67-positive cells [ΔKi-67 (%)] was calculated as (Ki-67 [%] on day 4)—(Ki-67 [%] average in retinol-untreated mice on day 4). Data are combined from four independent experiments. **(D)** Ear tissues were homogenized to isolate mRNA, and *Cxcl1* and *Cxcl2* expression was measured by quantitative RT-PCR analysis and normalized to that of *Actb*. Data are combined from four independent experiments. **(E)** The number of Ly6G^+^ CD11b^+^ neutrophils was determined on the basis of total cell numbers and flow cytometric data. **(F)** Ear thickness was measured on day 4. Ear swelling (Δµm) was calculated as (Ear thickness [µm] on day 4)—(Ear thickness [µm] before the first retinol application). Horizontal bars indicate median values. Data are combined from five independent experiments. *p < 0.05; **p < 0.01; ***p < 0.001.

To reveal the pathways associated with the anti-inflammatory properties of mead acid, we evaluated epidermal hyperplasia and keratinocyte proliferation in response to treatment with kinase inhibitors after retinol treatment. We found that the K10-staining layer of the epidermis and the area stained with K10 were decreased by treatment with SB202190 (a p38 MAPK inhibitor) and U0126 (a MAPK/ERK inhibitor), but not by treatment with SP600125 (a JNK inhibitor) (Figures 5A, B). In addition, the percentage increase in Ki-67-positive keratinocytes and the increases in expression of *Cxcl1* and *Cxcl2* on keratinocytes were reduced only by SB202190 treatment (Figures 5C, D). Consistent with this finding, SB202190 treatment after retinol application reduced the number of neutrophils at the site of inflammation (Figure 5E). Moreover, although U0126 treatment had no effects on keratinocytes (i.e., cell proliferation and chemokine production) (Figures 5C, D), its application decreased the numbers of neutrophils at the site of inflammation (Figure 5E). Reflecting these suppressive effects on keratinocyte and neutrophil activities, ear swelling was reduced by treatment with either SB202190 or U0126, but not by SP600125 treatment (Figure 5F).

Secretory granules from neutrophils contain proteases that induce further injury to, and activation of, keratinocytes. Our finding that the topical application of U0126 reduced neutrophil infiltration (Figure 5E) suggested that the MAPK/ERK pathway regulates neutrophil function and thus reduces keratinocyte hyperproliferation. In contrast, given that topical application of SB202190 reduced keratinocyte activities such as proliferation and chemokine production, we considered that the p38 MAPK pathway is essential for induction of the keratinocyte abnormalities induced by retinol. In addition, we confirmed that further inhibition of keratinocyte abnormalities such as enhancement of keratinocyte proliferation and production of neutrophil chemoattractants, and ear swelling was not observed upon co-treatment with SB202190 and mead acid (Supplementary Figures S2A–C). These results suggested that the p38 MAPK pathway is a candidate for the target pathway regulated by mead acid.

### Mead acid inhibits p38 MAPK activity in HaCaT cells

**FIGURE 6:**
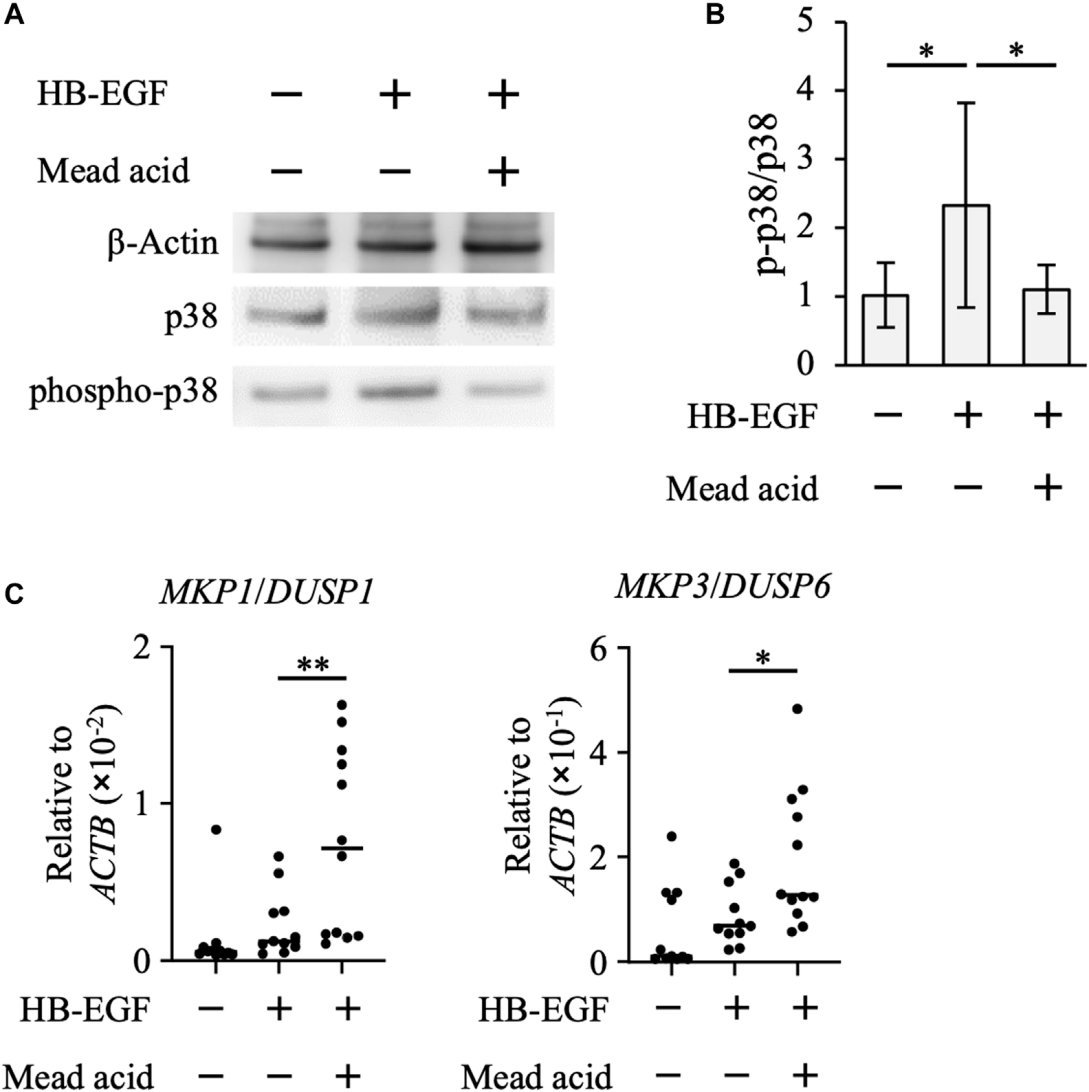
Mead acid attenuates p38 MAPK activation. HaCaT cells were pretreated with mead acid or vehicle before stimulation with HB-EGF (1 ng/mL). **(A)** The expression levels of phospho-p38, p38, and β-actin were measured by western blotting. Representative images from three independent experiments are shown. **(B)** The ratio of phospho-p38 to p38 was calculated by dividing the intensity of expression of phospho-p38 by the intensity of that of p38. **(C)** The expression levels of *MKP1*/*DUSP1* and *MKP3/DUSP6* were measured by quantitative RT-PCR analysis and normalized to that of *ACTB*. Data are combined from four independent experiments. *p < 0.05; **p < 0.01.

By using the human keratinocyte cell line HaCaT, we next examined whether mead acid inhibited the p38 MAPK pathway. HB-EGF is an inducer of keratinocyte hyperplasia, and *Hbegf* expression on keratinocytes is enhanced through RAR and RXR heterodimer activation, which is activated by retinol (Rittie et al., 2006). We found that mead acid did not reduce *Hbegf* expression on keratinocytes after retinol treatment (Supplementary Figure S3), suggesting that the inhibitory effect of mead acid was downstream of HB-EGF signaling. Therefore, we next analyzed the phosphorylation of p38 by stimulating HaCaT cells with HB-EGF for 30 min after mead acid treatment. Western blot analysis showed that the expression level of p38 was not changed by mead acid treatment, whereas the expression of phospho-p38 was significantly reduced by mead acid treatment (Figures 6A, B). To identify the mechanism of reduction of phospho-p38, we focused on two enzymes that catalyze the phosphorylation of p38 and are known to activate p38 MAPK; and protein phosphatases that catalyze dephosphorylation and are negative regulators of phospho-p38 (Kidger and Keyse, 2016). We confirmed that the expression levels of the MAPK kinases *MKK3* and *MKK4* were not affected by mead acid treatment after retinol application (Supplementary Figure S4). The largest group of protein phosphatases that specifically regulates MAPK activity in mammalian cells is the dual-specificity phosphatase (DUSP) family of phosphatases, which are known as MAPK phosphatases (MKPs) (Kidger and Keyse, 2016; Chen et al., 2019). We next evaluated *MKP* gene expression on HaCaT cells after stimulation with HB-EGF. Mead acid upregulated the expression of *MKP1*/*DUSP1* and *MKP3*/*DUSP6* compared with vehicle treatment (Figure 6C). These results suggested that mead acid resolves p38 phosphorylation by enhancing *MKP* expression in keratinocytes, consequently reducing p38 MAPK activation.

## Discussion

We reported previously that mead acid directly regulates neutrophil migration and LTB_4_ production, consequently inhibiting DNFB-induced allergic contact hypersensitivity (Tiwari et al., 2019). Here, we newly showed the anti-inflammatory activity of mead acid in retinol-induced ICD through its regulation of not only neutrophil functions but also keratinocyte functions *via* a PPARα-mediated pathway. We further found that retinol-induced activation of p38 MAPK is crucial to the induction of keratinocyte abnormalities and is downregulated by mead acid.

Fatty acid metabolites act as endogenous ligands for nuclear receptors such as RXRs and PPARs, and they have beneficial effects on skin inflammation. For example, 12-hydroxyeicosapentaenoic acid, an eicosapentaenoic acid-derived metabolite, suppresses DNFB-induced allergic contact hypersensitivity by inhibiting the production of the neutrophil chemoattractants Cxcl1 and Cxcl2 *via* RXRα (Saika et al., 2021). In addition, αKetoA (10-oxo-*cis*-12-*cis*-15-octadecadienoic acid), an α-linolenic acid metabolite produced by intestinal bacteria, inhibits DNFB-induced allergic contact hypersensitivity by inhibiting the nuclear translocation of NF-κB (nuclear factor kappa-light-chain-enhancer of activated B cells) in macrophages *via* PPARγ (Nagatake et al., 2022). Here, we evaluated the PPAR ligand activity of mead acid in reporter assays, and we showed that mead acid is a potent ligand of PPARα and PPARβ/δ; however, only PPARα, not PPARβ/δ, is functional in ameliorating retinol-induced ICD.

PPARα ligands have been widely shown to have anti-inflammatory effects by regulating the keratinocyte activation observed in various types of dermatitis, including irritant and allergic contact dermatitis, atopic dermatitis, and ultraviolet (UV)-induced erythema (Sheu et al., 2002; Dubrac and Schmuth, 2011; Shin et al., 2016). The anti-inflammatory activities of PPARα ligands on skin diseases have been observed in humans (Kippenberger et al., 2001). In addition, PPARα activation is involved in the regulation of cell proliferation and inflammatory chemokine and cytokine production. For example, PPARα activation by clofibrate inhibits cell proliferation in *vivo* and *in vitro* studies (Komuves et al., 2000). In addition, topical application of WY-14643 (pirinixic acid), a specific PPARα ligand, reduces the expression of the pro-inflammatory cytokines IL-1β and IL-6 and attenuates skin inflammation in atopic dermatitis (Staumont-Sallé et al., 2008). Consistent with these previous reports showing anti-inflammatory effects through PPARα activation, we showed here that mead acid regulates keratinocyte abnormalities *via* a PPARα-mediated pathway in retinol-induced ICD and is a promising candidate for suppression of the side effects of retinol treatment. In contrast, PPARβ/δ activation promotes keratinocyte proliferation and is a key event in hyperproliferation in psoriasis, because *Hbegf i*s one of the direct target genes of PPARβ/δ (Romanowska et al., 2008). In addition, PPARα production was decreased in dermatitis lesions, whereas PPARβ/δ production, which is upregulated by central mediators of the inflammatory response, such as tumor necrosis factor alpha and interferon gamma, is increased in the epidermis in atopic dermatitis and psoriasis lesions compared with in normal areas of the skin (Westergaard et al., 2003; Romanowska et al., 2008). This difference in the gene expression patterns of PPARα and PPARβ/δ during the course of dermatitis indicates that PPARβ/δ activation is related to the exacerbation of inflammation. Our finding that mead acid decreases retinol-induced ICD *via* PPARα is consistent with previous reports indicating that the functions of the two isoforms, PPARα and PPARβ/δ, do not entirely overlap (Icre et al., 2006), and PPARα activation is a promising efficacious candidate for reducing retinol-induced ICD. In addition, because PPARα ligands are being found to have diverse health effects in hyperlipidemia, Alzheimer’s disease, and alcoholism, mead acid may be a promising tool for alleviating not only skin diseases but also a variety of inflammatory diseases (Hack et al., 2012; Beckenbach et al., 2015; Pawlak et al., 2015).

The signals required for keratinocyte hyperproliferation in retinol-induced ICD have not been elucidated, but in psoriasis cutaneous p38 MAPK activation is a crucial event for the development of clinical signs, including epidermal thickening (Sakurai et al., 2019). We found here that p38 MAPK is also a major downstream signaling pathway for the induction of the keratinocyte abnormalities induced by retinol stimulation, such as keratinocyte hyperplasia and inflammatory chemokine expression. In addition, our *in vitro* studies using HaCaT cells suggested that the downregulation of p38 MAPK caused by mead acid application was led by increases in the expression of the MAPK phosphatases *MKP1* and *MKP3*, which play essential roles in tuning the activity of MAPK family members. In particular, MKP1 inactivates p38 MAPK more efficiently than it does MAPK/ERK, whereas MKP3 inactivates MAPK/ERK rather than p38 MAPK signaling (Owens and Keyse, 2007; Kidger and Keyse, 2016). Several reports have shown the involvement of MKPs in the reduction of inflammatory mediator production and inflammatory responses. For example, 12-hydroxyheptadecatrienoic acid upregulates *MKP* expression and suppresses p38 MAPK activation, thereby reducing the expression of *IL-6* in UV-B-irradiated HaCaT cells (Lee et al., 2012). In addition, mice deficient in MKP1 overproduce pro-inflammatory cytokines, and MKP1 knockout mice are highly susceptible to imiquimod-induced psoriasis, in which enhancement of p38 phosphorylation and increased *Cxcl1* and *Cxcl2* expression are also observed (Zhao et al., 2018). Attenuation of p38 MAPK activation and reduction of inflammation are also likely to occur at least in part *via* MKP3 enhancement, because MKP3 overproduction inhibits lipopolysaccharide-induced p38 phosphorylation and reduces inflammation in human umbilical vein endothelial cells (Unenkhuu et al., 2021). These reports support the hypothesis that upregulation of MKP1 and MKP3 reduces keratinocyte hyperproliferation and chemokine production in retinol-induced ICD *via* p38 MAPK attenuation. These results suggest that the crosstalk between PPARα activation and p38 MAPK downregulation can be explained in terms of MKP function. However, the PPARα ligand 5,8,11,14-eicosatetraenoic acid increases MKP1 mRNA stability by inducing HuR (human antigen R), an RNA-binding protein known to enhance mRNA stability, and suppresses chemokine expression in primary astrocytes *via* a PPARα-independent pathway (Lee et al., 2012). Therefore, we need to study the interaction between PPARα activation and MKP upregulation in more depth.

Mead acid regulates not only keratinocyte function but also neutrophil activation to reduce LTB_4_ production by neutrophils and neutrophil recruitment to sites of inflammation by reducing the pseudopod formation promoted by fMLP (*N*-formyl-methionyl-leucyl-phenylalanine) administration (Tiwari et al., 2019). PPARα activation reduces LTB_4_ production and the further acceleration of LTB_4_ metabolism (Narala et al., 2010). As neutrophils express PPARα, mead acid may directly inhibit LTB_4_ production by neutrophils and further recruitment of neutrophils *via* the PPARα-mediated pathway. We showed here that mead acid reduced p38 MAPK activation, which is essential for the promotion of keratinocyte abnormalities. In addition, we found that the numbers of neutrophils at sites of inflammation were decreased not only by treatment with SB202190, but also by U0126. However, evaluation of neutrophil chemotaxis by using a p38 MAPK inhibitor has shown that the neutrophils are still capable of migration toward fMLP, but not as effectively as untreated neutrophils (Kim and Haynes, 2013). Indeed, in the presence of a p38 MAPK inhibitor, neutrophils show decreased expression of adhesion molecule surface markers such as CD11b and CD66b and of chemoattractant receptors such as formyl peptide receptor 2, LTB_4_-receptor, and CXC chemokine receptor 1, which are needed to recognize chemoattractant and neutrophil adhesion to endothelial cells (Kim and Haynes, 2013). These reports indicate that a p38 MAPK-dependent pathway plays a role in neutrophil chemotaxis, in part through the regulation of surface marker and receptor expression. In contrast, the MAPK/ERK pathway plays an important role in promoting neutrophil mobility (Ichiki et al., 2016), and it plays a key role in transducing “go” signals to neutrophils, not only during their emigration from venules but also during their migration within tissues (Mizuno et al., 2014). In addition, MAPK/ERK activation of endothelial cells is required for neutrophil infiltration (Stein et al., 2003). Consistent with these findings, we showed that treatment with the MAPK/ERK inhibitor U0126 reduced ear swelling and neutrophil numbers at sites of inflammation without reducing the hyperproliferation of keratinocytes or the gene expression of neutrophil chemoattractants on keratinocytes; it did, however, reduce retinol-induced ICD. Because MKP3 strongly inhibits MAPK/ERK activation, it is likely to be responsible for the direct effects of mead acid on neutrophils (Kidger and Keyse, 2016). Therefore, mead acid likely directly regulates neutrophil function by attenuating the MAPK/ERK pathway or the p38 MAPK pathway, or both, by enhancing *MKP1* and *MKP3* expression. However, in our study it was not clear whether MAPK/ERK activation in neutrophils was attenuated by mead acid treatment. In addition, the relationships among PPARα, MAPK/ERK and p38 MAPK, and neutrophil function, and the mead-acid-stimulated function of these molecular signals in neutrophils, have not yet been revealed. Future research needs to reveal precisely how neutrophil functions are regulated by mead acid.

In summary, we showed here that mead acid reduced retinol-induced ICD by inhibiting keratinocyte hyperproliferation and gene expression of the neutrophil chemoattractants *Cxcl1* and *Cxcl2 via* a PPARα-mediated pathway. In addition, we revealed that mead acid downregulated p38 MAPK activation in HaCaT cells by upregulating the expression of *MKP1* and *MKP3*. We indicated that topical application of mead acid can reduce the ICD observed as a side effect of retinoid treatment. Topical administration of mead acid appears promising for improving retinoid therapy.

## Data Availability

The raw data supporting the conclusion of this article will be made available by the authors, without undue reservation.

## Supplementary material

SUPPLEMENTARY FIGURE S1 Mead acid and oleic acid are not toxic to HaCaT cells. HaCaT cells (1 × 10^4^ cells) were incubated with mead acid and oleic acid adjusted to the indicated final concentrations for 1 h and **(B)** 24 h. The percentage of viable cells was calculated by using the following formula: (%) = [100 × (sample OD_570_)/(vehicle control OD_570_)]. Data are combined from two independent experiments, *n* = 7 or 8. There were no significant differences in cell viability between the mead acid and oleic acid treatments at each concentration or among the concentrations of either acid.

SUPPLEMENTARY FIGURE S2 Mead acid does not have further inhibitory effects upon co-treatment with p38 MAPK inhibitor. Irritant contact dermatitis was induced by topical application of retinol for 4 consecutive days. Mice topically received p38 MAPK inhibitor or vehicle (1:1 DMSO and 50% [v/v] ethanol in PBS). Mead acid or vehicle (75% [v/v] ethanol in PBS) was then applied. **(A)** Ear thickness was measured on day 4. Ear swelling (Δµm) was calculated as (Ear thickness [µm] on day 4)—(Ear thickness [µm] before the first retinol application). **(B)** Ear samples were obtained on day 4. The percentage of Ki-67-expressing keratinocytes gated as CD45^−^ CD31^−^ CD34^−^ CD49f^+^ was measured by flow cytometry. The increase rate of Ki-67^+^ cells [ΔKi-67 (%)] was calculated as (Ki-67 [%] on day 4)—(Ki-67 [%] average in retinol-untreated mice on day 4). **(C)** Ear tissues were homogenized to isolate mRNA, and *Cxcl1* and *Cxcl2* expression was measured by quantitative RT-PCR analysis and normalized to that of *Actb*. Data are combined from four independent experiments. Horizontal bars indicate median values. NS, not significant; **p* < 0.05; ***p* < 0.01; ****p* < 0.001; *****p* < 0.0001.

SUPPLEMENTARY FIGURE S3 Mead acid does not reduce *Hbegf* expression on keratinocytes in mice. Mice were pretreated with mead acid or vehicle (75% [v/v] ethanol in PBS) for 30 min before stimulation with retinol or vehicle. Eight hours later, CD45^−^ CD31^−^ CD34^−^ CD49f^+^ keratinocytes were isolated, and then mRNA was extracted to perform quantitative RT-PCR analysis. *Hbegf* expression, which was normalized to that of *Actb*, was measured. Data are combined from two independent experiments. Horizontal bars indicate median values. NS, not significant.

SUPPLEMENTARY FIGURE S4 Mead acid does not inhibit *MKK3* and *MKK4* expression on keratinocytes. HaCaT cells (2 × 10^6^ cells) were pretreated with mead acid or vehicle for 30 min before stimulation with HB-EGF (1 ng/mL) for 1 h. The expression levels of *MKK3* and *MKK4* were measured by quantitative RT-PCR analysis and normalized to that of *Actb*. Data are combined from four independent experiments. Horizontal bars indicate median values. NS, not significant.
