# Supplementary figures and images for: Mead acid inhibits retinol-induced irritant contact dermatitis via peroxisome proliferator-activated receptor alpha
Source: Front Mol Biosci. 2023 Feb 7;10:1097955. doi: 10.3389/fmolb.2023.1097955 (PMC9941550; doi:10.3389/fmolb.2023.1097955)

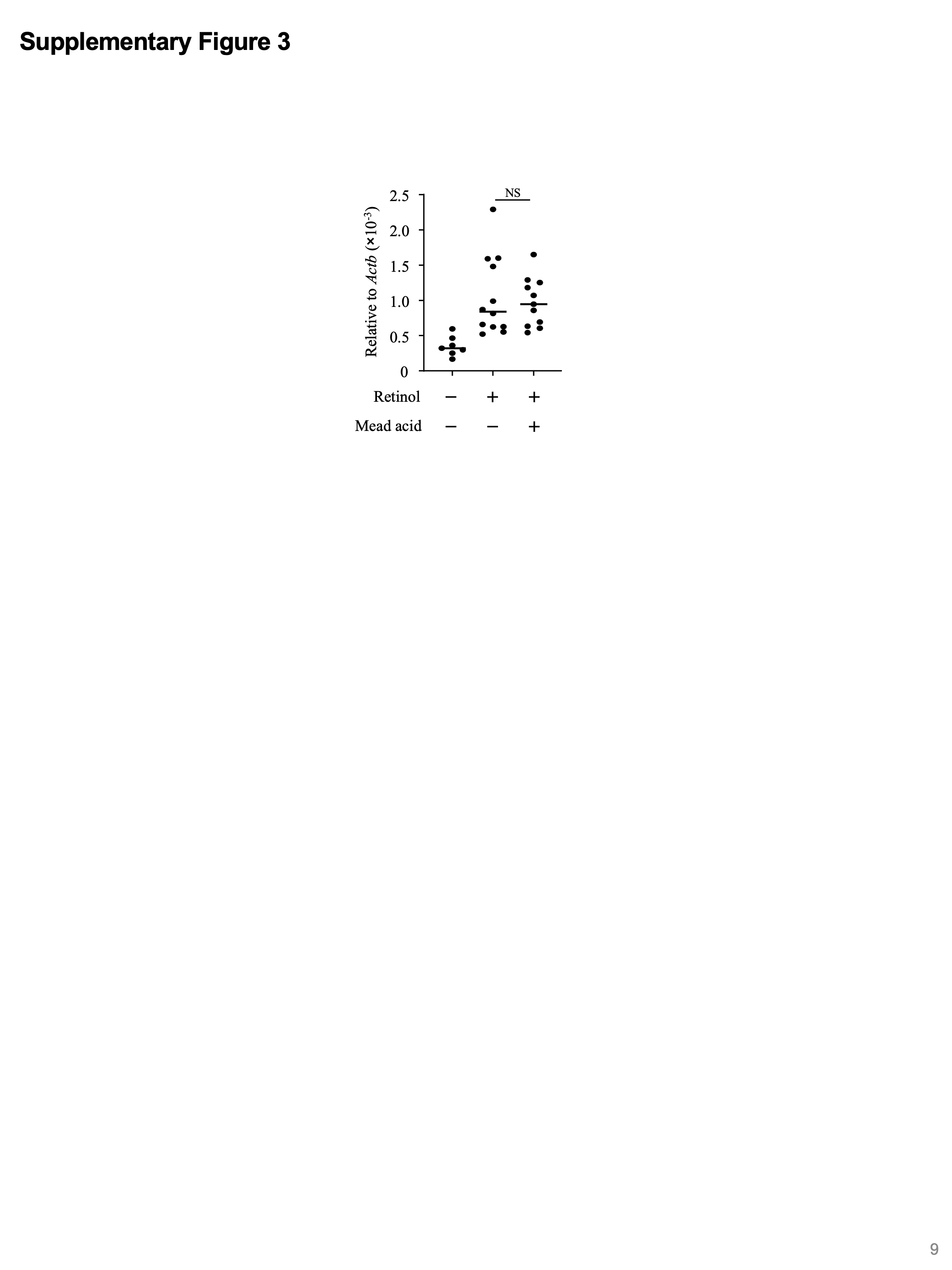

Supplement: Supplementary file 1 [file Image3.TIFF]

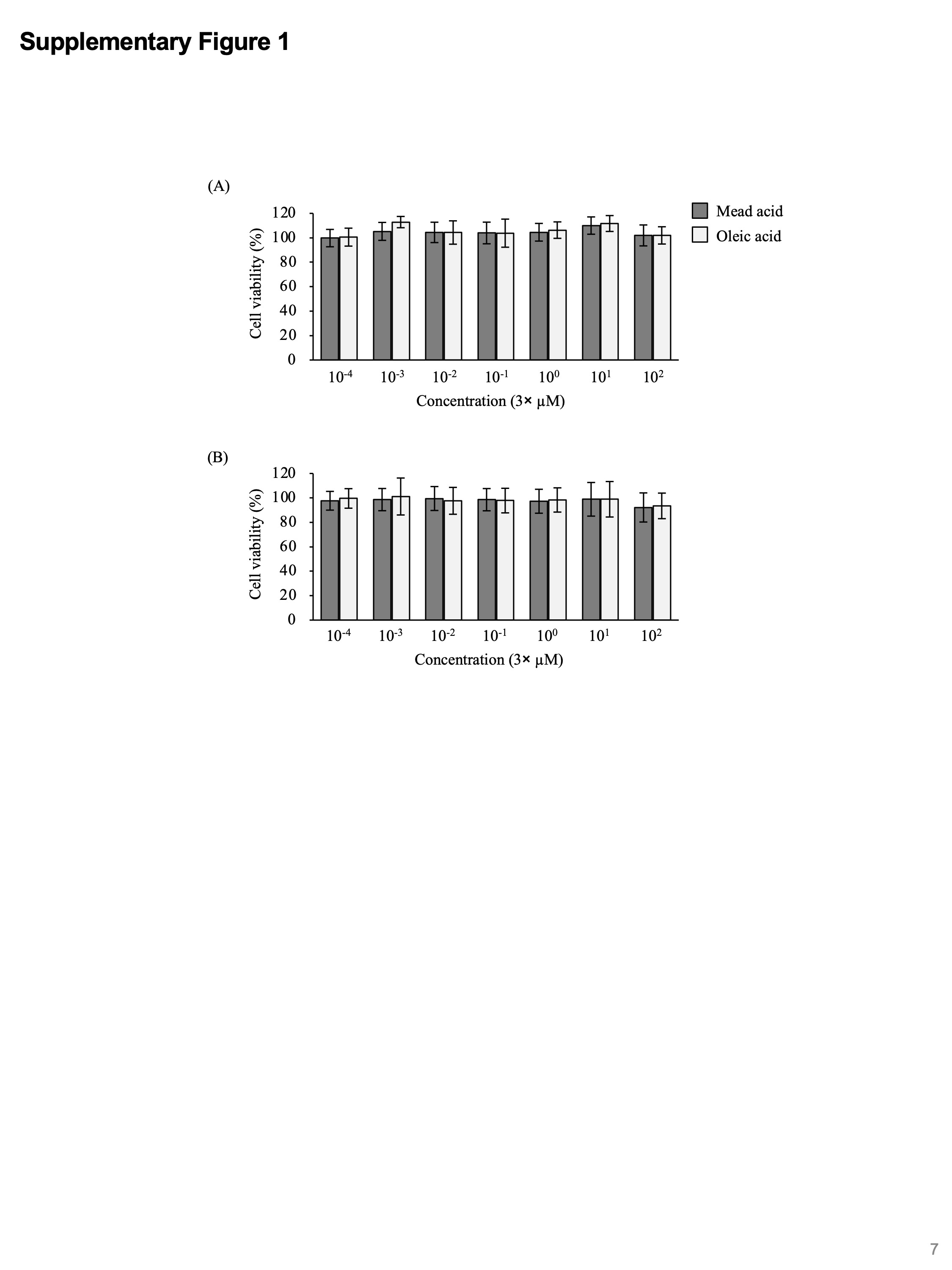

Supplement: Supplementary file 2 [file Image1.TIFF]

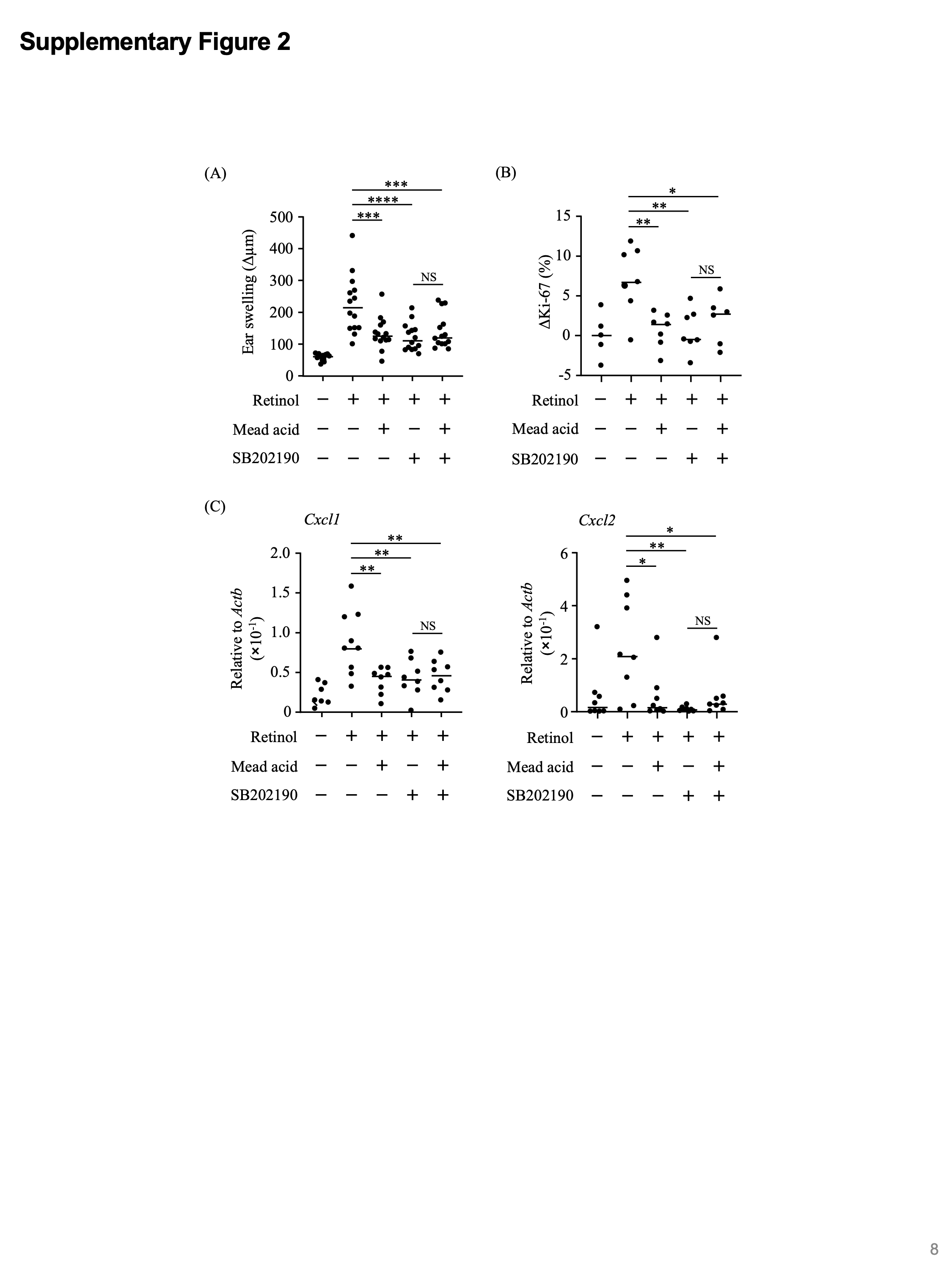

Supplement: Supplementary file 3 [file Image2.TIFF]

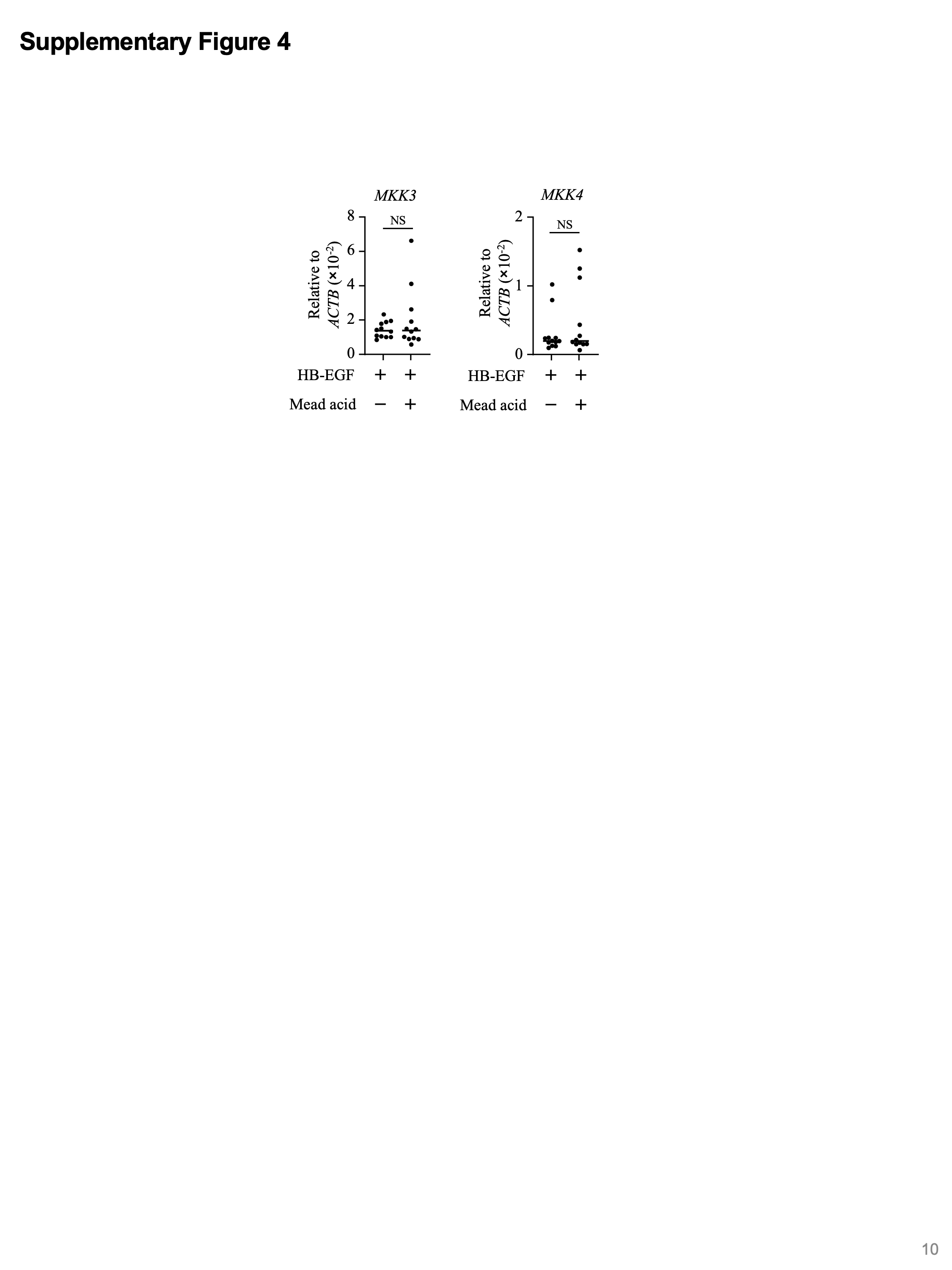

Supplement: Supplementary file 4 [file Image4.TIFF]
